# Caseinate-Stabilized Emulsions of Black Cumin and Tamanu Oils: Preparation, Characterization and Antibacterial Activity

**DOI:** 10.3390/polym11121951

**Published:** 2019-11-27

**Authors:** Lucie Urbánková, Věra Kašpárková, Pavlína Egner, Ondřej Rudolf, Eva Korábková

**Affiliations:** 1 Department of Fat, Surfactant and Cosmetics Technology, Faculty of Technology, Tomas Bata University in Zlín, nám. T. G. Masaryka 5555, 760 01 Zlín, Czech Republic; 2 Centre of Polymer Systems, Tomas Bata University in Zlín, nám. T. G. Masaryka 5555, 760 01 Zlín, Czech Republic

**Keywords:** black cumin (*Nigella sativa*) seed oil, tamanu (*Calophyllum inophyllum*) seed oil, emulsion, formulation, antibacterial activity

## Abstract

Caseinate-stabilized emulsions of black cumin (*Nigella sativa*) and tamanu (*Calophyllum inophyllum*) oils were studied in terms of preparation, characterization, and antibacterial properties. The oils were described while using their basic characteristics, including fatty acid composition and scavenging activity. The oil-in-water (o/w) emulsions containing the studied oils were formulated, and the influence of protein stabilizer (sodium caseinate (CAS), 1–12 wt %), oil contents (5–30 wt %), and emulsification methods (high-shear homogenization vs. sonication) on the emulsion properties were investigated. It was observed that, under both preparation methods, emulsions of small, initial droplet sizes were predominantly formed with CAS content that was higher than 7.5 wt %. Sonication was a more efficient emulsification procedure and was afforded emulsions with smaller droplet size throughout the entire used concentration ranges of oils and CAS when compared to high-shear homogenization. At native pH of ~ 6.5, all of the emulsions exhibited negative zeta potential that originated from the presence of caseinate. The antibacterial activities of both oils and their emulsions were investigated with respect to the growth suppression of common spoilage bacteria while using the disk diffusion method. The oils and selected emulsions were proven to act against gram positive strains, mainly against *Staphylococcus aureus* (*S. aureus*) and *Bacillus cereus* (*B. cereus*); regrettably, the gram negative species were fully resistant against their action.

## 1. Introduction

In recent years, there has been increasing demand for the use of biopolymers as emulsion stabilizers in the cosmetic, pharmaceutic, and food industry. Additionally, alternative sources of bioactive substances, such as essential fatty acids, are in focus. Many of the natural products that were extracted from plants demonstrate biological activities, and they receive particular attention as a source of valuable fatty acids, antimicrobials, antioxidants, or wound healing agents [1,2].

*Nigella sativa*, which is commonly called black cumin (BC), and *Calophyllum inophyllum* (tamanu, TA), have been used for its nutritional and therapeutic value for many years. Black cumin and its seeds with a characteristic strong taste have been exploited for both culinary and medicinal purposes. The reason for the biological activity of cumin seeds (antioxidant, anticancer, anti-inflammatory, as well as antibacterial) can be found in their complex composition, as they contain over 100 different constituents, including all essential fatty acids. In general, BC consists of oil, proteins, and carbohydrates [2,3]; oil from black cumin is rich in linoleic and oleic acids, as well as bioactive phytosterols and tocopherols [4]. In addition, the seeds contain active phytochemicals, such as thymoquinone, thymohydroquinone, q-cymene, carvacrol, and 4-terpineol [5]. This oil can be used as a natural resource for the production of pharmaceuticals and novel functional foods thanks to its composition and effects [2,6,7,8].

Tamanu oil expelled from the seeds of *Calophyllum inophyllum* is mainly beneficial in the treatment of dermal problems. The antibiotic and anti-inflammatory properties make this oil an excellent raw material for regenerating and protective formulations. Calophyllic acid and a lactone with antibiotic properties are the two main bioactive substances in the tamanu oil [9]. *Calophyllum inophyllum* is also known as a rich source of secondary metabolites (coumarins, xanthones, flavonoids, and triterpenes) [10], with some of them having anti-inflammatory, antibacterial, and antiviral properties [11,12]. Additionally, coumarin derivatives (calaustralin, calophyllolide, inophyllum, inophyllum E) that were obtained from a crude extract of the nuts were reported to have activity against *Staphylococcus aureus* [1] and antioxidant properties [13].

Nevertheless, the presence of unsaturated fatty acids in both oils causes their sensitivity to oxidation. This makes encapsulation a suitable strategy for protecting the oils, thus allowing for the delivery of lipophilic compounds into aqueous-based systems. For the efficient encapsulation, the production of stable emulsions is crucial and the properties of stabilizing surface layer, oil, as well as the emulsion characteristics are the central factors that can affect the performance of final product; emulsification also plays a key role in optimizing the encapsulation efficiency of oils [14,15].

Emulsification of triacylglycerol-based oils (fish, olive, or sunflower oil) is frequently conducted with milk proteins [16,17,18,19,20]. Particularly, sodium caseinate (CAS) is a common emulsifier and it can be the integral part of formulations due to its amphiphilic structure and surface activity. In o/w emulsions, CAS has been used as emulsifier and encapsulating agent [21], as it forms a barrier by adsorbing to the oil-water interface. This barrier is essential for protecting the bioactive substances against oxidation and the molecules that are adsorbed at oil-water interface also provide an effective shield against flocculation and coalescence due to a combination of electrostatic and steric repulsion. For example, CAS provided good protection of fish oil against oxidation by forming a physical barrier effect [16,22,23,24]. Casein is also able to protect other oils against oxidation. As published by Hu, et al., corn o/w emulsions that were stabilized with CAS showed both high physical and oxidative stability, which was probably due to ability of CAS to produce a thick layer on emulsion droplet interface and its unique chelating properties [25].

The above summary clearly shows that the incorporation of rarely studied tamanu and black cumin oils in emulsions can be of benefit, as they contain active ingredients with biological and pharmaceutical effects (treatment of asthma, bronchitis, skin diseases; antimicrobial, anti-inflammatory effects; and, gastro-protective properties) [8,9,26,27]. Moreover, the stabilizing of emulsions containing these oils with biocompatible and biodegradable protein CAS can facilitate the delivery of oils to hydrophilic systems while also protecting them against degradation. Therefore, the aim of the work was to prepare o/w emulsions of tamanu and black cumin oil stabilized with CAS, which can serve as carrier systems with therapeutic and physiological benefits for humans. The study also aimed at finding the optimum emulsion formulation with respect to the concentrations of both CAS and oils, and at establishing a suitable emulsification procedure when using two commonly available methods, high-shear homogenization and sonication. The novelty of the study lies in the determination of antimicrobial properties of the oils and their emulsions against both gram positive and gram negative strains, as contamination with spoilage microorganisms is one of the crucial problems encountered in cosmetic and food industry. In the view of the fact that the studies that deal with the emulsification of black cumin and tamanu oils are relatively scarce and only limited data on this topic is found in the scientific literature, the presented study is original and it brings about topical information in this area.

## 2. Materials and Methods

### 2.1. Materials

Casein sodium salt (CAS), DPPH (1,1-diphenyl-2-picrylhydrazyl), toluene, and sodium thiosulfate were provided from Sigma-Aldrich (Darmstadt, Germany). Non-traditional vegetable oils from *Calophyllum inophyllum* (Tamanu oil, TA) and *Nigella sativa* (Black cumin oil, BC) were obtained from Nobilis Tilia (Krásná Lípa, Czech Republic). All of the chemicals and reagents were of analytical grade and used without further purification.

### 2.2. Characterization of Oils

#### 2.2.1. Basic Characteristics

Iodine value (IV; Hanus) expressing the amount of unsaturation in fatty acids present in the oil, saponification value (SV), which is a measure of average molecular mass of all fatty acids, acid value (AV) corresponding to amount of free fatty acid in the oil, and peroxide value (oxidation stability) were determined while using volumetric analysis. The standard methods that were performed with slight modifications were followed [28].

#### 2.2.2. Fatty Acid Composition by Gas Chromatography-Flame Ionization Detector (GC-FID)

The composition of fatty acids in oils was determined by GC-FID after their conversion to respective methyl esters (FAME). In the case of tamanu oil, 30 mL methanol, 2 mL methanolic phenolphthalein solution, and 5 mL hexane were used to dissolve 2 g oil. The solution was heated for approx. 15 min. and the hot sample was then titrated with 0.5M methanolic KOH to neutralize the sample. The sample was added 1 mL 1M methanolic potassium hydroxide and then heated again for 30 min. After the reaction was completed, the FAMEs were twice extracted with 2 × 10 mL hexane. FAME of black cumin oil were prepared via mixing of 2 g oil with 20 mL methanol and 0.5 mL methanolic KOH (1 M). The solution was heated for 30 min. and the resulting products were twice extracted with 2 × 10 mL hexane. The samples of methyl esters were diluted with hexane for analysis.

GC analyses were conducted while using a DANI Master GC Fast Gas Chromatograph (DANI Instruments, Cologno Monzese, Italy) that was equipped with a flame ionization detector (FID) (DANI Instruments, Cologno Monzese, Italy) and capillary column Phenomenex Zebron^TM^ ZB-5MS (30 m × 0.25 mm × 0.5 μm, Phenomenex Inc., Torrance, CA, USA). The injection volume was of 1 μL and flow of a nitrogen carrier gas of 1 mL·min^−1^. The temperature gradient started at 110 °C, followed by increase of 5 °C·min^−1^ to 240 °C and then to 280 °C at the rate of 2 °C·min^−1^. The sample was held at the temperature for 20 min. The temperatures of injector and detector were set to 230 and 300 °C, respectively. The FAMEs were identified by comparing their retention times with those of *SUPELCO^TM^ 37 Component FAME Mix* standard (Sigma Aldrich, Darmstadt, Germany). The quantification of FAME was performed according to internal normalization method. The results are reported in percentage (wt %) of respective fatty acid/100 g total fatty acid content.

#### 2.2.3. Antioxidant Activity

Free radical scavenging activity of BC and TA oils was assayed according to [4]. The DPPH radicals were dissolved in toluene at concentration of 10^−4^ M. For evaluation, 10 mg of oil (in 100 µL toluene) was mixed with 390 µL toluenic DPPH^•^ solution and then vortexed for 20 s. The decrease in absorbance at 515 nm was measured after 1, 15, 30, 45, and 60 min. of mixing while using a Photolab 6600 UV-VIS photometer (Xylem Analytics Germany Sales GmbH & Co. KG, WTW, Weilheim, Germany) with toluene as the blank. The scavenging activity of the samples was calculated while using the equation *I* = (*A*_0_ − *A*_1_)/*A*_0_ × 100 (%), where *I* is inhibition activity, *A*_0_ is the absorbance of the blank and *A*_1_ is the absorbance of the mixture.

#### 2.2.4. Interfacial Tension

The interfacial tension of oil-water systems was determined by the pendant drop technique while using an Attension Theta optical tensiometer (Biolin Scientific, Gothenburg, Sweden). The images of the droplets were recorded with a black and white digital camera and the surface tension was obtained by iterative fitting of the shape of the droplet with the Young-Laplace Equation. The droplet was formed while using a 0.718 mm (22 gauge) stainless steel needle.

### 2.3. Preparation of Emulsions

The o/w emulsions were formulated with 1, 2, 5, 7.5, 10, or 12 wt % CAS. The CAS solutions were prepared by dispersing the powder in deionized water under gentle stirring at room temperature for 4 h. The sample was left to stand at 4 °C overnight to allow for complete hydration and, prior to emulsification, the solution was allowed to equilibrate to room temperature under stirring. Emulsifications were carried out by mixing each of the above CAS solutions and oil phase (5, 10, 20, and 30 wt %; TA or BC oil) at 25 °C. The pH of CAS solutions was not adjusted prior to emulsification.

Two different methods were carried out to prepare the emulsions: a) a high-shear homogenization with an rotor-stator device Ultra-Turrax T25 (IKA, Staufen, Germany) for 12 min. at 13, 400 rpm and b) an ultrasonic homogenization (UP400S, Hielscher Ultrasonics, Teltow, Germany) with 400 W, 24 KHz, for 1 min., operated with 100% amplitude. The total sample volume was of 20 mL. Emulsions were kept in the ice bath during sonication. After emulsification, the pH of prepared emulsions was measured while using pH meter (CPH 51, Elteca, Turnov, Czech Republic) and values of 6.44 ± 0.03 and 6.45 ± 0.04 were recorded for TA and BC oil, respectively.

### 2.4. Characterization of Emulsions

#### 2.4.1. Size of Emulsion Droplets and Zeta Potential

The size and distribution of emulsion droplets were measured while using the laser diffraction instrument Mastersizer 3000 (Malvern Instruments, Malvern, UK) that was capable of measuring within the size range of 0.01 to 3500 μm. The emulsions were suspended in recirculating milliQ-water flowing through the Hydro SM measuring cell of the instrument, at a pump velocity of 2, 400 rpm. The volume moment mean diameter *D*_(4,3)_ was determined by instrument software according to equation *D*_(4,3)_ = *Σni*·*d*^4^/*Σ ni*·*d*^3^, where *n_i_* is the number of droplets and *d* represents the droplet diameter. The value of *D*_(4,3)_ corresponds to a mean diameter of spheres with the same volume as the analysed droplets. The emulsions were also observed with optical microscope Zeiss AxioCam MR 5 (Carl Zeiss MicroImaging GmbH, Oberkochen, Germany). Prior to observation, a droplet of emulsion (10 μL) was placed onto a glass microscope slide and then viewed under 10–100× magnification.

The zeta potential of the samples was measured while using a Zetasizer Nano (Malvern Instruments, Malvern, UK) and calculated using the Smoluchowski model. For measurements, 5 µL of the sample were diluted with 1 mL of twice filtered (Millipore, 0.22 µm) deionized water. The average of three records on the freshly prepared samples is reported.

#### 2.4.2. Creaming Index

The stability of emulsions was assessed by visual observation, daily during the first week of preparation and then at seven-day intervals. The emulsions were stored at ambient temperature. The stability was expressed as the height of emulsion layer (*H_emu_*_l_), relative to the total height of the emulsion (*H_total_*), which is referred to as the creaming index (*CI*); (*CI* = *H_emul_*/*H_total_* × 100 (%)).

### 2.5. Antimicrobial Activity

The antimicrobial activities of oils and emulsions were evaluated while using eight bacterial strains that were obtained from the Czech Collection of Microorganisms (CCM, Brno, Czech Republic). The bacteria were selected to represent major food-borne classes. The gram positive (G^+^; *Micrococcus luteus* CCM 732, *Staphylococcus aureus* subps. *aureus* CCM 3953, *Bacillus cereus* CCM 2010, *Enterococcus faecalis* CCM 2665) and gram negative (G^-^; *Escherichia coli* CCM 3954, *Pseudomonas aeruginosa* CCM 3955, and *Salmonella enterica* subsp. *enterica ser. Enteritidis* CCM 4420, *Serratia marcescens* subsp. *marcescence* CCM 303) strains were employed in the test. All of the microorganisms were maintained on nutrient agar and sub-cultured onto fresh media every two weeks. Suspensions of bacterial strains were prepared by inoculation from pure culture on a Petri plate into sterile tube with nutrient broth and incubation at 30 °C for 24 h (*Pseudomonas aeruginosa* and *Bacillus cereus*). Other bacterial strains were incubated at 37 °C for 24 h.

The antimicrobial activities of oils and emulsions were determined while using the disc diffusion assay. An overnight culture of each of the bacterial strains in nutrient broth was adjusted to 10^6^ CFU/mL and 100 µL of inoculum was spread onto Mueller-Hinton Agar (MHA) sterile agar plates (Hi-Media Laboratories, Mumbai, India). Sterile paper discs (5 mm in diameter) were soaked with 7 µL of oil or with 5 µL of emulsion and then placed on the inoculated agar. As references, discs that were soaked with sterile deionized water and 7.5 wt % aqueous CAS dispersions were used. All of the inoculated plates were incubated at 37 °C for 24 h for mesophilic bacterial strains and at 30 °C for 24 h for *Pseudomonas aeruginosa* and *Bacillus cereus.* After incubation, the antimicrobial activity of the tested samples was evaluated by measuring the diameter of inhibition zone (mm). The diameter of the disc was subtracted from the diameter of inhibition zone.

### 2.6. Statistical Analysis

All the analyses were conducted at least in triplicates, with the Dean-Dixon method being utilized to calculate the means and standard deviations. The Student T-test was applied to detect any statistical differences between the samples (Statistica, StatSoft, Inc., Palo Alto, CA, USA). The P (probability) value of ≤0.05 was considered to be statistically significant.

## 3. Results and Discussion

### 3.1. Properties and Antioxidant Activity of Oils

#### 3.1.1. Basic Characteristics and Fatty Acid Composition

To evaluate differences between the oils, their properties were determined by measuring their basic characteristics, namely iodine (IV), saponification (SV), acid (AV), and peroxide (PV) values, and via determining their fatty acid composition (Table 1 and Table 2). The SV and PV were similar for both oils and they are in agreement with the previously published values. The AVs of TA oil were significantly higher (43.6 ± 0.6 mg KOH·g^−1^) in comparison with those of BC oil (8.4 ± 0.7 mg KOH·g^−1^) and they roughly corresponded to the values that were reported in literature [29,30]. In this respect, it can be also stressed that the acid value of TA oil was notably higher than that of the common vegetable oils, thus giving evidence of an increased amount of free fatty acids in this oil. Both of the oils showed high SV, which is a measure of the average molecular mass of fatty acid in the oil. The IV values indicated a higher degree of unsaturation for cumin oil, which was also confirmed by GC analyse, showing a higher content of linoleic acid C18:2 in the sample (Table 1).

The composition of fatty acids in TA oil proved the presence of oleic acid C18:1 (41 wt %) as the dominating fatty acid, followed by linoleic acid C18:2 (28 wt %); stearic C18:0 (16 wt %) and palmitic C16:0 (14 wt %) acids were the major saturates. The minor amounts of C16:1, C17:0, and C20:0 were also detected. In BC oil, linoleic acid C18:2 (57 wt %) was the prevailing fatty acid, followed by oleic C18:1 (25 wt %), palmitic C16:0 (13 wt %), and stearic C18:0 (3.4 wt %) acids (Table 2). The results complied well with data regarding fatty acid composition of the oils reported in literature [29,31,32].

#### 3.1.2. Radical Scavenging Effect of Oils

**Figure 1:**
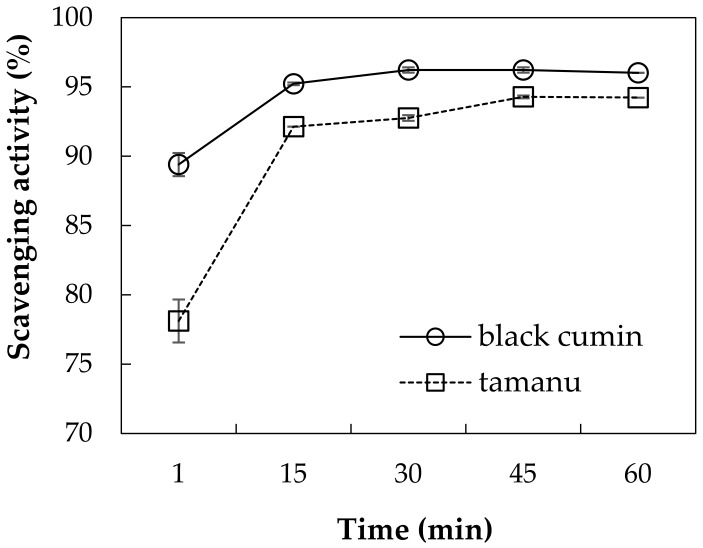
1,1-diphenyl-2-picrylhydrazyl (DPPH) free radical-scavenging activities and their development in time determined for BC and TA oils after dissolving in toluene.

The DPPH free radical scavenging assay is frequently used to estimate the antioxidant capacity of the substances. In current work, the time development of scavenging effects of BC and TA oils on DPPH^•^ was assayed in toluene and illustrates a slightly higher activity of cumin oil than that of TA oil (Figure 1). After 60 min. of incubation with radicals, 96% of DPPH^•^ were quenched by cumin oil, while TA oil was able to inhibit 94% radicals. The oils were already efficient after 1 min. of incubation time, with 89 and 78% activity for TA and black cumin oil, respectively. The current results are somewhat higher than those that were reported by [4], who used the same procedure and found out that after 60 min. of incubation, 60% of radicals were inhibited by BC seed oil. The scavenging action of both oils can be mainly ascribed to (1) the content and composition of unsaponifiables, (2) the diversity in structural characteristics of phenolic antioxidants present, (3) the synergism of the phenolic antioxidants with other bioactive components, and (4) the different kinetic behaviours of potential antioxidants [4].

### 3.2. Emulsion Properties, Influence of Processing Conditions, and Composition

#### 3.2.1. Droplet Size and Distribution

The size of emulsion droplets is an important parameter with a crucial effect on emulsion stability. Light diffraction analyses showed that the emulsion droplet size (*D*_(4,3)_) was influenced by all of the studied variables, especially by the method of preparation and concentration of stabilizing CAS; and, to lesser extent, by the type and content of both oils.

**Figure 2:**
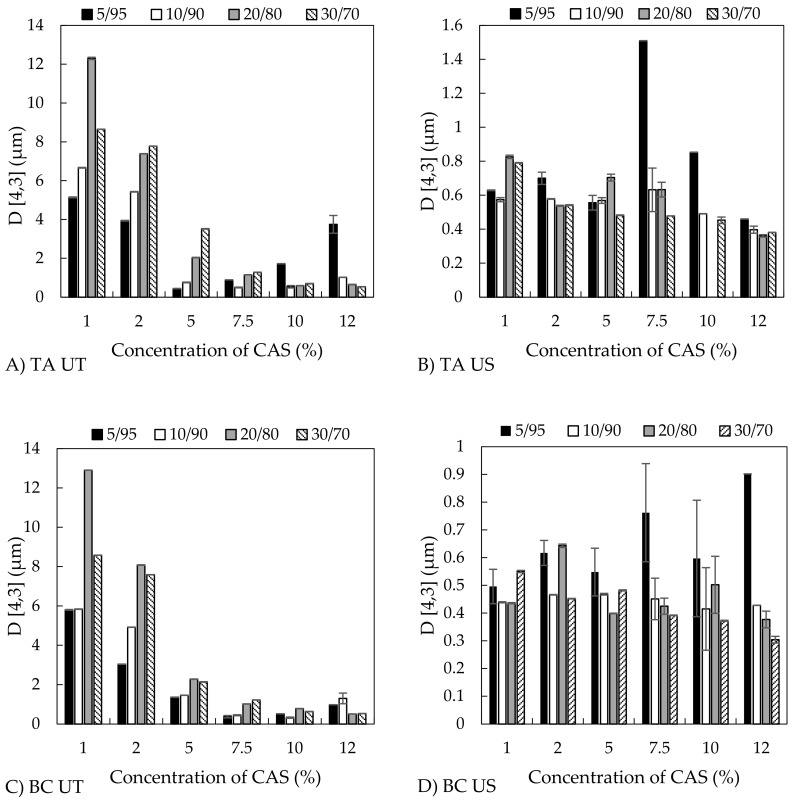
Influence of casein sodium salt (CAS) concentration and o/w ratio on volume weighed diameter of emulsion droplets (*D*_(3,4)_) of (**A**) TA oil emulsions prepared by Ultra-Turrax (UT) (**B**) TA oil emulsions prepared by sonication (US) (**C**) BC oil emulsions prepared by UT, and (**D**) BC oil emulsion prepared by US.

In this study, the emulsions were prepared by two different emulsification procedures, which involved high-shear homogenization (Ultra-Turrax, UT) or sonicator (US), which obviously controlled the size of emulsion droplets. Emulsification with UT led to coarse emulsions with bigger droplets that ranged from 0.3 to 13 µm, with their properties being notably affected by composition, such as o/w ratio and CAS content (Figure 2A,C). On the other side, the homogenization with US yielded fine emulsions, with *D*_(4,3)_ varying from 0.3 to 0.9 µm and 0.4 to 1.5 µm for BC and TA emulsions, respectively; hence, with a sufficiently small size that is a prerequisite for production of stable systems (Figure 2B,D) [33]. Although droplets after sonication were notably smaller than droplets that were treated with Ultra-Turrax, some differences between emulsions containing BC and TA oils were observed. These can be explained by the character, composition, and physical properties of the oils, which affect the emulsifying efficiency and hence the droplet size of emulsions. Here, an important role can play two crucial characteristics, namely viscosity of the oil and interfacial tension at oil-water interface. The viscosities of the used oils are notably different, TA oil is viscous (20–26 mPa·s) [34], whilst the viscosity of BC oil is lower (6.3 mPa·s) [35]. On the other hand, both of the oils have low and rather similar interfacial tensions at the oil-water interface, and values of 8.4 ± 1.0 mN·m^−1^ and 4.3 ± 0.5 mN·m^−1^ were measured for BC and TA oil, respectively. Therefore, the larger droplets of TA emulsions are likely due to the higher viscosity rather than the differences in the interfacial tension between oils. Interestingly, Wooster et al. also referred that high-viscosity oils formed emulsions with larger droplets than oils with low viscosity [36].

**Figure 3:**
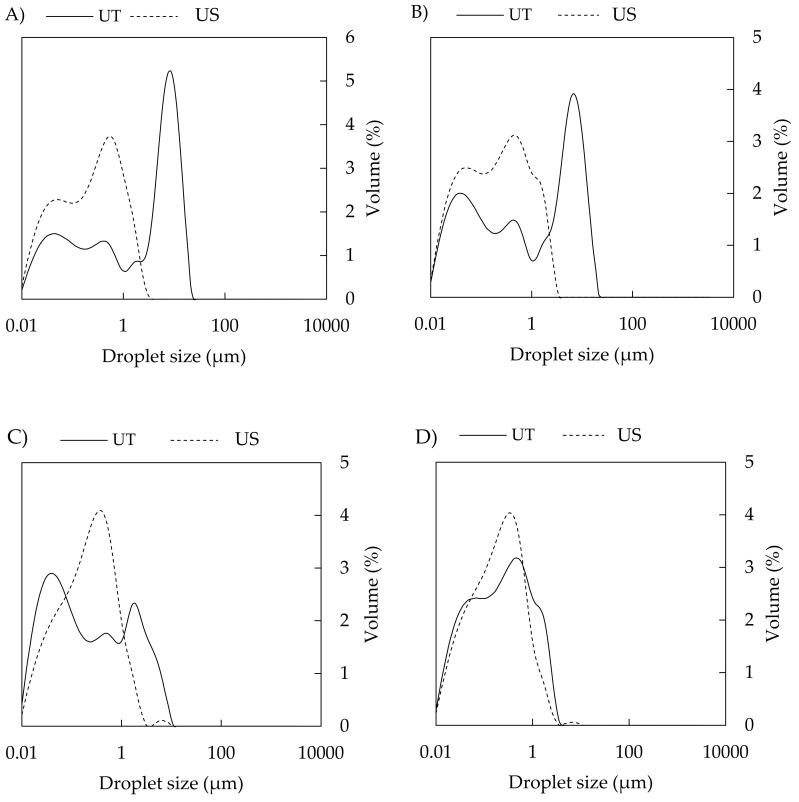
Influence of homogenization method on distribution curves of CAS stabilized emulsions. Ultra-Turraxed (UT) and sonicated (US) emulsions are compared across oil type, o/w ratio and CAS concentrations: (**A**) BC emulsions with 2 wt % of CAS (10/90); (**B**) TA emulsions with 5 wt % of CAS (30/70); (**C**) BC emulsions with 7.5 wt % of CAS (20/80); and, (**D**) BC emulsions with 12 wt % of CAS (20/80).

In addition, the droplet distributions in US emulsions were narrower when compared to those that were prepared with UT (Figure 3). This finding is in agreement with studies that were performed on emulsions stabilized by other types of biopolymers, such as CAS/polysaccharides [17,37], whey protein concentrate [38], or on emulsions containing modified starch and maltodextrin [39].

**Figure 4:**
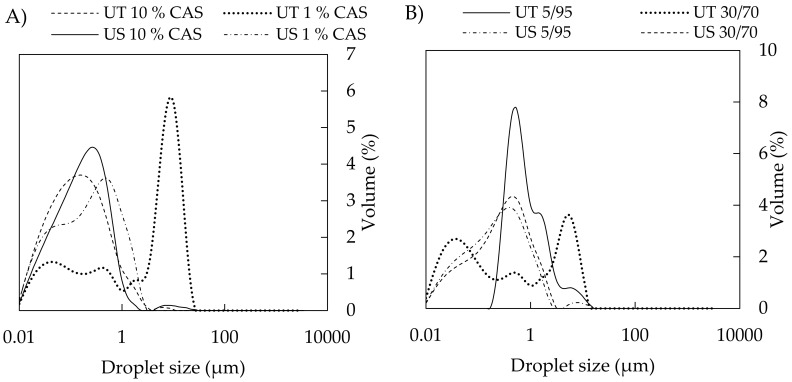
(**A**) Effect of CAS concentration on distribution curves of BC emulsions with o/w ratio 10/90 prepared by UT and US; (**B**) effect of o/w ratio on distribution curves of BC emulsions with 5 wt % CAS. For tamanu oil, the trend is similar (data not shown).

The influence of the processing method on droplet size must always be considered in combination with a composition of emulsions. Regarding the concentration of CAS, the droplet size of UT-prepared emulsions gradually decreased with increasing protein concentration (1 to 5 wt %); however, *D*_(4,3)_ was significantly reduced as CAS concentrations increased to 7.5–12 wt % (Figure 2A,C). Full coverage of droplets with protein occurs at a CAS content of approximately 3 wt % (emulsions with 35 % tetradecane oil phase) [40]. At a higher protein concentration (4 wt %), a higher amount of CAS than required for saturation coverage of the oil droplets is presented; hence, a range of CAS concentrations used in current work is sufficient for completely covering the arising droplets. Therefore, the bigger droplets that were prepared by UT (in the case of sufficient CAS available in the system) can be assigned to the discrepancy between the higher rate of coalescence of oil droplets being formed by a given energy input and the lower rate of CAS adsorption at the oil-water interface during homogenization. Under these conditions, the uncovered droplets tend to come together and form larger droplets again. On the other hand, when the CAS content in aqueous phase is insufficient, flocculation might occur due to casein bridging between droplets, also resulting in rapid creaming [17,20,41,42]. The results from particle sizing were also supported by the distribution curves of UT-emulsions, where a shift in droplet diameter toward smaller sizes was observed as the protein concentration increased, and distribution curves changed from multimodal (1 wt % CAS) to monomodal (10 wt % CAS), which indicated the formation of a stable emulsions (Figure 4A) [43].

**Figure 5:**
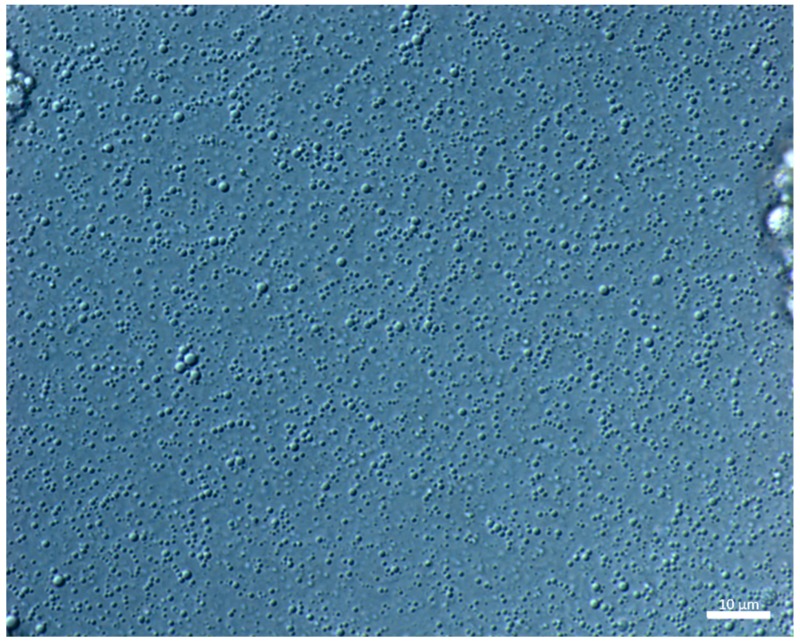
Optical microscopy (Magnification 20x) of BC emulsions with o/w 20/80 prepared with 1 wt % CAS while using sonication. Scale bar is 10 µm.

On the contrary, emulsions that were prepared by US contained much smaller droplets, and only minor changes in the droplet size alongside change in CAS concentration were observed (Figure 2B,D). For emulsions that were prepared with the aid of US, the distribution curves were mostly monomodal with the simultaneous shift in direction to lower droplet sizes (Figure 4A). Figure 5 visualizes an example of light microscopy figure captured on the emulsion prepared with US.

The volume fraction of oil is another parameter with an impact on emulsion droplet size. In the current study, the increasing oil content influenced (*D*_(4,3)_); however, only at lower concentrations of CAS (1–7.5 wt %) and mainly in the case of UT-prepared emulsions. Here, the droplet size increased with an increasing oil fraction. The effect is documented in Figure 4B, showing the broad distribution curve with three droplet populations being recorded for UT emulsions containing 30 wt % BC oil and 5 wt % CAS; on the other hand, by lowering the oil content to 5 wt %, monomodal distributions were obtained. CAS contents of 10 and 12 wt % afforded droplets that were notably smaller and influence of oil fraction was only marginal. In contrast, the situation was different when it came to emulsions that were prepared by sonication (Figure 4B) and only minor changes in *D*_(4,3)_ and distributions with the increasing oil content were observed at the entire concentration range of CAS used. In systems containing high concentrations of both protein and oil, the emulsions can be stabilized through the formation of a weak particle-based gel network, which slowly reorganizes under the influence of gravity and internal osmotic stresses [40].

Alongside with other variables, the type of oil plays also a role with respect to the droplet size in emulsions, and the droplets of the BC emulsions that were prepared by UT were smaller as compared to droplets containing the TA oil. As already discussed above, the differences can be explained by composition and physical properties of the oils, mainly viscosity and interfacial tension. This difference was again wiped off in emulsions that were prepared using US. The fact that TA oil also contains, in addition to neutral lipids and glycolipids, phospholipids (1.6%), small amounts of sterols, and mono- and diacylglycerols, which all have emulsifying properties, can also contribute to easier formation of smaller droplets [9].

#### 3.2.2. Zeta Potential

**Figure 6:**
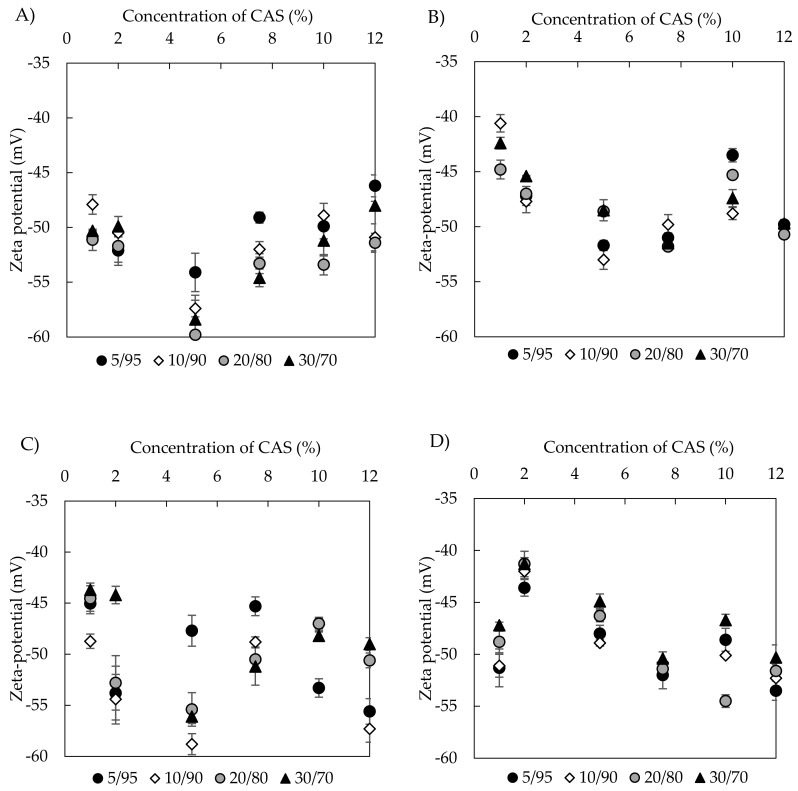
Zeta potential of freshly prepared emulsions at native pH of ~6,5 as a function of o/w ratio and CAS content (**A**) BC emulsions prepared with Ultra-Turrax (UT), (**B**) BC emulsions prepared with sonication (US), (**C**) TA emulsions prepared with UT, and (**D**) TA emulsions with US.

Zeta (ξ) potential is commonly considered as a stability indicating parameter of dispersion systems. However, the values of ξ potential alone are not always capable of predicting the stability of emulsions, mainly those that are sterically stabilized. Immediately after preparation, the ξ potential ranged from −53 to −41 mV for emulsions with BC oil (prepared with US) and from –60 to –46 mV for those that were prepared with UT. TA emulsions behaved similarly with the potential values lying from –55 to −41 mV and –59 to −44 mV for US and UT emulsions, respectively. In general, UT treatment seemed to deliver slightly lower ξ potentials and, obviously, no or minor systematic influence of oil content on the potential was observed. However, CAS concentrations above 5 wt % might have a positive influence in potential lowering, mainly for emulsions with TA oil (Figure 6). The negative charges of emulsion droplets are, therefore, due to the presence of CAS stabilizer. As such, this protein is negatively charged at a natural pH and its aqueous dispersion in the absence of oil showed a potential of −18.7 mV.

In current study, ξ potential measurements were conducted on emulsions with non-adjusted pH (TA oil emulsions pH = 6.44 ± 0.03, BC oil emulsions pH = 6.45 ± 0.04). At a pH close to caseinate isoelectric point (pH 4.6), the CAS emulsions are unstable [44], thanks to a reduction in electrostatic repulsion between the droplets. However, at pH 6, the negative ξ potential of CAS assured emulsion stability due to electrostatic and steric stabilization mechanisms.

Differences in the ξ potentials between emulsions that were prepared by UT and US can be assigned to combined effect of sonication treatment and oil properties. When compared with many other proteins, caseins are particularly disordered and substantially hydrophobic, which assist in their rapid adsorption to oil droplet surfaces during emulsification. In aqueous solutions, CAS forms a random coil with only small amount of secondary structure due to the relatively high content of hydrophobic amino acids (proline residues) [45]. During sonication, the hydrophobicity of CAS changes, thanks to the different orientation of proteins at the oil-water interface, which can impact the ξ potential of emulsions formed by US treatment [46]. The impact of oil type on ξ potential, which is observed in our work, was also reported for soy bean protein isolate emulsions containing medium chain triacylglycerols, palm, soybean, and rapeseed oils after US treatment [47]. The lowering of ξ potential was ascribed to the formation of protein aggregates and different amounts of protein adsorbed onto emulsions containing studied oils of different compositions.

#### 3.2.3. Phase Studies and Emulsion Stability

When proteins are used to stabilize emulsions, the main mechanism governing their creaming in the presence of non-adsorbed stabilizer is related to depletion flocculation [48]. Kinetic stability of emulsions, as described by creaming index (CI), provides indirect information on droplet flocculation and destabilization occurring in an emulsion.

**Figure 7:**
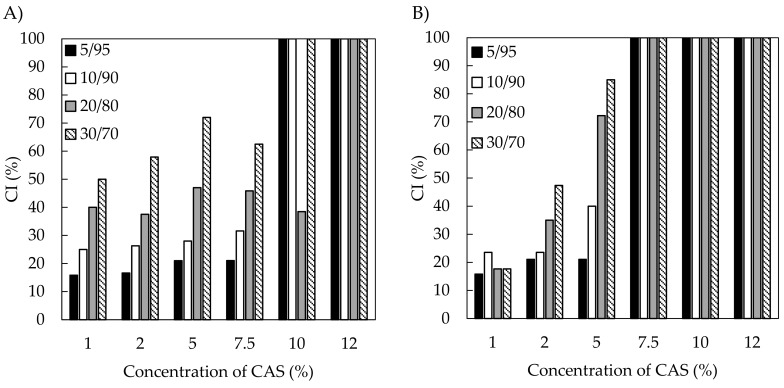

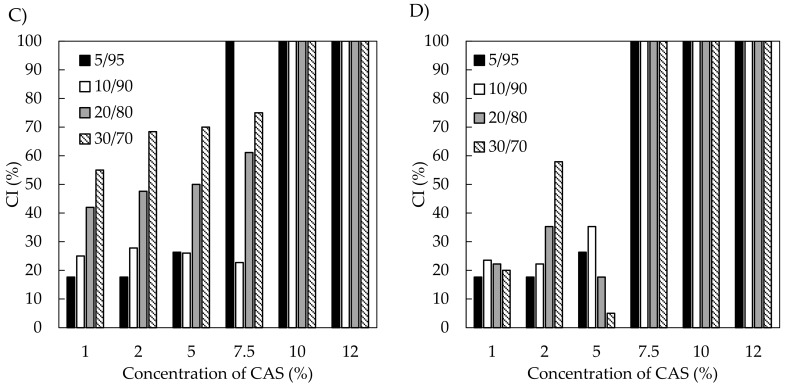
Comparison of creaming index (CI; CI—100% is for stable emulsion) determined on freshly prepared emulsions, as affected by processing method and composition of emulsions (**A**) BC emulsions prepared with Ultra-Turrax (UT), (**B**) BC emulsions prepared with sonication (US), (**C**) TA emulsions prepared with UT, and (**D**) TA emulsions with US.

Not all of the emulsions prepared in the current study demonstrated kinetic stability, even immediately after preparation. When observed by naked eye, some of them formed a bottom serum layer and a small cream phase on the top. On the other hand samples with specific formulations, mainly with high CAS content, exhibited good resistance to creaming. Figure 7 shows the CI of the freshly prepared emulsions. Stable emulsions (CI = 100%) were observed at CAS concentrations of 10–12 wt % (UT emulsion) and 7.5–12 wt % CAS (US emulsions). However, samples containing lower concentrations of CAS destabilised readily and the extent of destabilization, expressed as CI, was obviously dependent on oil content. More specifically, the CI increased with an increasing amount of oil, which was more notable on UT emulsions in comparison with emulsions that were prepared by US. Therefore, the formation of stable emulsions was considered to be a synergic effect of the US processing, resulting in small droplets with narrower distribution and the sufficient amount of CAS. Only a minor difference in CI was observed between emulsions with TA or BC oils.

The long-term stability of the emulsions was evaluated by the recording of changes in their visual appearance during storage until the point of emulsion breaking appeared. Not surprisingly, the most stable emulsions were those that contained the highest protein content (12 wt % CAS). For example, BC emulsions with 30 wt % oil prepared by both procedures remained unchanged for seven days of storage with CI being of 100%. Stability studies also corroborated the fact that US treatment assured the production of emulsions with limited creaming. At a CAS concentration of 10 wt %, the emulsions that were manufactured with US were stable towards creaming for seven days, whilst in emulsions that were prepared with UT, the creaming already started three days after preparation. Moreover, UT manufactured emulsions with even lower CAS content were prone to breakdown and underwent phase separation approximately after a week of storage. Interestingly, in most emulsions, although the CI value was lower immediately after emulsification, it did not notably change during their storage. From the CI data, it was also apparent that the creaming process took about a week to be completed, thereafter compaction of the cream layer started, which stopped when no more oil droplets and proteins could be packed in the top cream layer. The compaction/compression of the cream layer was also reported by [49]. These results can be generalized for both BC and TA emulsions. The above observations demonstrate that an important role in stability of CAS-containing emulsions is related to concentration of this biopolymer in the aqueous phase, which closely correlates with emulsion viscosity. The viscosity of CAS emulsions is controlled by the interactions between the droplets and mainly by the nature and strength of interparticle attractive forces, which depends on the structure of the CAS layer that was adsorbed at the oil-water interface. Additionally, the CAS self-assembly and aggregation of non-adsorbed CAS in the aqueous phase play an important role with respect to the viscosity and stability behaviour of CAS stabilized emulsions [50].

**Figure 8:**
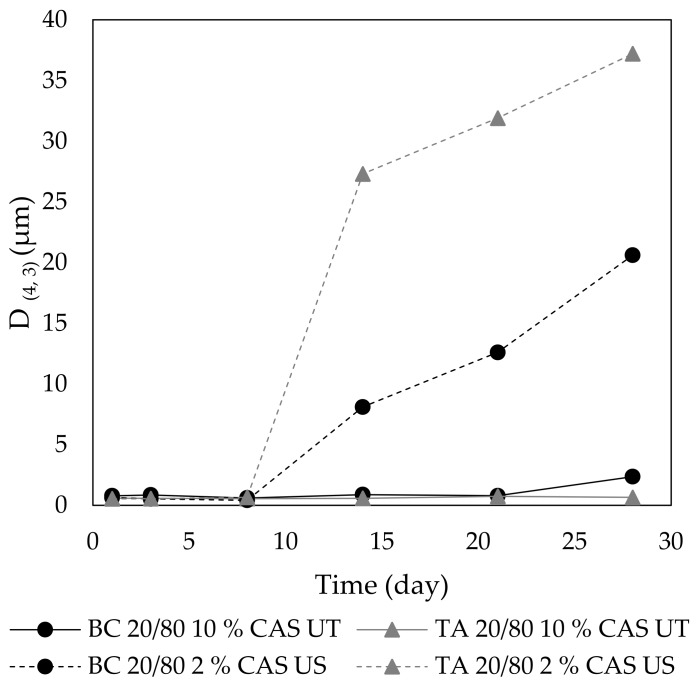
Long term stability of TA and BC emulsions stabilized with CAS determined at room temperature: development of volume weighed diameter of emulsion droplets (*D*_(4,3)_) in time.

The data from the observation of creaming were in reasonably good correlation with particle sizing measurements, which were also conducted during emulsion storage. The results revealed that the *D*_(4,3)_ increased during a month-long storage at an ambient temperature. The US emulsions changed their droplet sizes to higher extent than the UT systems; however, emulsions that were prepared with the aid of UT were more prone to phase separation and their breakdown occurred earlier. Here again, the key role played the content of CAS in emulsions. Emulsions with 1 or 2 wt % CAS were stable in terms of size only during the first days, emulsion with higher CAS content for a week and emulsions with 10 and 12 wt % CAS remained unchanged for a longer period of time (Figure 8). This is in agreement with findings in [51]. However, when considering the practical application of TA and BC emulsions, their long-term stability is not satisfactorily. In this respect, the prolongation of sonication time or two-stage homogenization with an additional treatment of coarse emulsions with high-pressure homogenizer can be used for the improvement of stability. The effect of high-pressure homogenization on properties of CAS emulsions studied authors [33]. They compared properties of thus prepared emulsions with emulsions that were prepared by Ultra-Turrax and concluded that UT produced larger droplets and less stable systems than the homogenizer did. However, during homogenization, the choice of correct pressure was essential, as the over-processing of emulsions and conformation changes of proteins can occur at higher pressures [52].

### 3.3. Antimicrobial Activity

Illnesses that are caused by pathogenic bacteria and/or their toxins are of great concern to public health. For the suppression of microorganism growth, plant extracts with antimicrobial activity can also be used [4].

Antibacterial activity of oils and emulsions (o/w 30/70) prepared thereof with 2 and 7.5 wt % CAS was determined while using the disc diffusion method and expressed in terms of the size of the inhibition and halo zones (mm).

**Figure 9:**
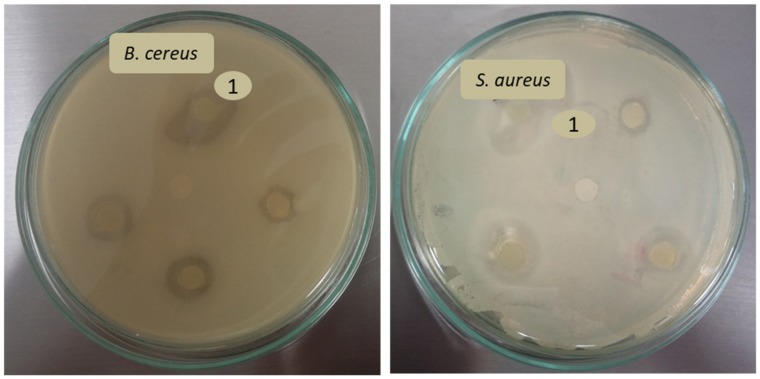
Images of growth media with inhibition zones observed after application of TA and BC emulsions: from “1” clockwise: TA oil with 2 wt % CAS, BC oil with 2 wt % CAS, TA oil with 7.5 wt % CAS, BC oil with 7.5 wt % CAS; in the middle is negative control.

Disc diffusion assay proved the inhibitory activity of both oils and selected emulsions against the gram positive bacteria, which was controlled by the type of the oil and the formulation of the emulsion. Regrettably, none of the samples were capable of suppressing the growth of the tested gram-negative species (*Escherichia coli, Pseudomonas aeruginosa, Salmonella enterica,* and *Serratia marcescens*). Table 3 shows that the effect of TA oil was significantly higher (p ≥ 0.5) than that of BC oil, both for *B. cereus* and *S. aureus*. It can be seen from the bigger inhibition zones with the sizes ranging from 8.2 ± 0.4 mm (*B. cereus*) to 6.0 ± 1.2 mm (*S. aureus*). This oil also caused lower bacterial growth (14.8 ± 1.3 to 7.7 ± 0.7 mm) around the inhibition zones (halo zone) and it was able to act, at least partially, against *M. luteus* and *E. faecalis* (halo zone only). The effects of TA oil encapsulated in emulsions with 2 and 7.5 wt % of CAS were rather similar, although the activity of emulsion stabilized with 2 wt % CAS was comparable with that of non-encapsulated TA oil (p ≥ 0.5). The antibacterial activity of the BC oil was, in comparison with TA oil, weaker, and this oil only showed antibacterial activity against *B. cereus* (5.7 ± 1.5 mm) and *S. aureus* (4.2 ± 0.7 mm). Emulsion that was stabilized with 2 wt % CAS containing BC oil exerted the comparable effect, which was weaker than that of BC oil alone. Neither BC oil nor its emulsions were efficient against *M. luteus* and *E. faecalis*. The example of growth media images with inhibition zones observed after application of TA and BC emulsions are shown in Figure 9. The lower activity of oils and emulsions against gram negative bacteria is a result of composition in their cell walls, as they contain an outer lipopolysaccharide membrane, which more efficiently protects the bacteria from the disruption caused by oils. As reported in literature, the antimicrobial activity of BC oil mainly results from the presence of thymoquinone, p-cymene, longifolene, and thymohydroquinone. Moreover, unsaturated, long chain fatty acids, such as linoleic and oleic acids present in the oil, were also reported to possess antibacterial and antifungal activity [8]. The study conducted by [4] supported the above results regarding the bioactivity of BC oil, also extending the range of its inhibitory activity to action against yeasts, namely *Saccharomyces cerevisiae* and *Candida albicans*. Regarding triacylglycerol-based TA oils, their bioactivity was thoroughly investigated by [1], who reported that TA oil exhibited two distinct antibacterial effects: the first against gram positive bacteria acting by direct inhibition of mitotic growth and the second potent effect against gram negative strains due to an increased release of β-defensin 2 peptide by macrophages. However, the results of our study have not confirmed the effect against gram negative strains.

## 4. Conclusions

In this work, sodium caseinate was used to stabilize emulsions containing bioactive tamanu and black cumin oils. The emulsions were prepared by ultrasound treatment or high-shear homogenization with Ultra-Turrax. The analysis of fatty acid composition in the oils revealed a higher degree of unsaturation for cumin oil with higher content of linoleic acid C18:2, which corresponds to the higher iodine value determined for this oil. Both of the oils effectively scavenged DPPH radicals, thus showing antioxidant activity. The results revealed that, under emulsification, sonication was the more efficient procedure and it afforded emulsions with a small particle size (0.3 to 1.5 μm) throughout the entire used concentration ranges of oils and caseinate. In addition to the emulsification technique, the ability to form stable emulsions of small, initial droplet sizes was mainly controlled by concentration of stabilizing caseinate and, to lesser extent, by the type and amount of used oils. In comparison with sonicated emulsions, the properties of emulsions that were prepared with Ultra-Turrax depended, to a higher extent, on their composition and the emulsions were more prone to destabilization. The oils and their selected emulsions exhibited antibacterial activity against gram positive strains (*S. aureus* and *B. cereus*); regrettably, the gram negative species were fully resistant against their action. Of the studied oils, the tamanu oil and its emulsions were more efficient. The antibacterial and antioxidant properties of the oils, together with their beneficial fatty acid composition, make them suitable for use as carriers for various lipophilic bioactive substances; the incorporation in caseinate emulsions further increases their applicability in hydrophilic systems.

## Figures and Tables

**Table 1:** Basic characteristics of tamanu (TA) and black cumin (BC) oils determined as iodine (IV), saponification (SV), acid (AV), and peroxide (PV) values.

Oil	IV (g I_2_/100 g)	SV (mg KOH·g^−1^)	AV (mg KOH·g^−1^)	PV (µval·g^−1^)
**TA**	83.3 ± 0.5	200.8 ± 1.9	43.6 ± 0.6	1.23 ± 0.0
**BC**	116.0 ± 0.9	197.2 ± 0.5	8.4 ± 0.7	1.05 ± 0.0

**Table 2:** Composition of fatty acids (wt %) in tamanu (TA) and black cumin (BC) oils determined by Gas Chromatography (GC).

		Fatty Acid (% of Total Content)						
Oil	C16:0	C16:1	C17:0	C18:0	C18:1	C18:2	C20:0	C22:0
**TA**	14.1 ± 0.3	0.3 ± 0.1	0.1 ± 0	15.7 ± 0.5	41.2 ± 0.2	27.6 ± 0.5	0.2 ± 0	0.2 ± 0
**BC**	12.6 ± 0.0	*n.d.*	*n.d.*	3.4 ± 0.6	24.9 ± 1.9	57.1 ± 2.6	*n.d.*	*n.d.*

*n.d.* not determined.

**Table 3:** Antibacterial activity of BC and TA oils and their 30/70 o/w emulsions against Gram positive strains; No inhibition zone towards *Micrococcus luteus* (*M. luteus*) and *Enterococcus faecalis* (*E. faecalis*) were observed.

Size of Zone ± SD (mm)						
	*B. cereus*		*S. aureus*		*M. luteus*	*E. faecalis*
	Inhibition	Halo	Inhibition	Halo	Halo	Halo
**Tamanu**						
**Oil**	8.2 ± 0.4	14.8 ± 1.3	6.0 ± 1.2	9.5 ± 1.3	9.3 ± 1.2	7.7 ± 0.7
**Emulsion** **2 % CAS**	7.8 ± 1.6	14.3 ± 0.7	6.0 ± 0.6	8.8 ± 0.4	14.3 ± 3.7	8.3 ± 1.4
**Emulsion** **7.5 % CAS**	6.3 ± 0.7	11.2 ± 1.3	4.7 ± 0.5	7.3 ± 0.5	9.0 ± 0.8	11.0 ± 3.1
**Black Cumin**						
**Oil**	5.7 ± 1.5	8.7 ± 0.9	4.2 ± 0.7	6.7 ± 0.9	*n.d*	*n.d*
**Emulsion** **2 % CAS**	2.0 ± 0.8	*n.d.*	3.3 ± 0.9	*n.d*	*n.d*	*n.d*
**Emulsion** **7.5 % CAS**	1.7 ± 0.5	*n.d.*	2.0 ± 0.6	*n.d*	*n.d*	*n.d*

*n.d.* not determined.
